# The cockroaches of *Balta* Tepper from China, with the description of four new species (Blattodea, Ectobiidae, Pseudophyllodromiinae)

**DOI:** 10.3897/zookeys.714.14041

**Published:** 2017-11-06

**Authors:** Zhi-Wei Qiu, Yan-Li Che, Yu-Hong Zheng, Zong-Qing Wang

**Affiliations:** 1 Institute of Entomology, College of Plant Protection, Southwest University, Beibei, Chongqing 400715, China

**Keywords:** Dictyoptera, distribution, key, new combination, new synonym

## Abstract

Four new species of cockroach genus *Balta* Tepper, 1893 are described and illustrated: *B. crena* **sp. n.**, *B. maculata* **sp. n.**, *B. tangi* **sp. n.**, and *B. yaoi* **sp. n.** *Balta picea* (Bey-Bienko, 1958) is now regarded as a new synonym of *Balta hwangorum* (Bey-Bienko, 1958), which is redescribed and illustrated. Two new combinations are proposed: *B. nodigera* (Bey-Bienko, 1958), **comb. n.** and *B. valida* (Bey-Bienko, 1958), **comb. n.**, and both species are redescribed and illustrated. A key to all species from China is provided.

## Introduction

*Balta* Tepper, 1893 is a large genus comprising 98 species, 40 of which are distributed in Australia, and 14 in Mainland China and Taiwan. The others are distributed in Pacific islands, Africa, and India. Bey-Bienko (1958) described three species of *Lupparia* Walker, 1868 and two species of *Balta* from China. Roth (1991) thought that *Balta* and *Lupparia* are quite similar. The genera *Balta* and *Lupparia* are distinguished by the size of the apical triangle of the hind wing (*Balta* spp. with smaller apical triangle). Che et al. (2010) briefly reviewed the research history of the genus; they also mentioned the relationship between *Balta* and *Lupparia* and some difficulty existing in discriminating these two genera. From then on, no one reported new species of the genus *Lupparia*. Recently, many cockroach specimens were received from Prof. Shuqiang Li (IOZCAS, Institute of Zoology, The Chinese Academy of Sciences), which were collected by means of canopy spraying conducted mainly in Yunnan and Hainan Provinces from 2009 to 2012. Material was also received from Prof. Shunxiang Ren and Zaifu Xu (SCAU, South China Agricultural University) that was collected from Guangdong Province.

After examining the specimens and comparing them with the original descriptions of Bey-Bienko (1958) and Asahina (1965), descriptions of four new species are appropriate, and are included here. Additionally, *Balta picea* (Bey-Bienko, 1958) is placed as a junior synonym of *Balta hwangorum* (Bey-Bienko, 1958), and two new combinations are proposed.

## Materials and methods

The terminology mainly follows Roth (2003). The terms for wing-veins are according to Li and Wang (2015). Morphological terms referring to spines are as follows: spines on the antero-ventral margin of the front femur with one or more proximal stout spines succeeded by a row of spinules of uniform length, terminating in two (B_2_) or three (B_3_) large spines (Type B); while the proximal stout spines absent (Type C) (Roth 2003). Genital segments of the examined specimens were macerated in 10% NaOH and observed in glycerin jelly using a Motic K400 stereomicroscope. All drawings were made with the aid of a Motic K400 stereomicroscope. Photographs of the specimens were taken using a Canon 50D plus a Canon EF 100mm f/2.8L IS USM Macro lens with the aid of Helicon Focus software. Material examined, including types of new species, is deposited in the Institute of Entomology, Southwest University (IESWU) in Beibei, Chongqing, China.

## Taxonomy

### Balta

Taxon classificationAnimaliaBlattodeaEctobiidae

Tepper, 1893

Balta Tepper, 1893: 39. Type species: Balta epilamproides Tepper, 1893: 39. Kirby 1904: 106; Shelford 1908: 19; Hebard 1943: 37; Princis 1951: 68; 1969: 968; Roth 1991: 967; Che et al. 2010: 56.
Mareta Bolívar, 1895: 371. Type species: Mareta conspicienda Bolívar; by monotype. Kirby 1904: 97; Shelford 1908: 10 (under Phyllodromia synonymy); Rehn 1922: 14; Hebard 1929: 18; Rehn 1931: 300. Synonymized with Balta by Princis 1969: 968.
Eoblatta Shelford, 1911: 155 (nec Eoblatta Handlirsch, 1906). Type species: Blatta notulata Stål, 1860; by monotype. Hebard 1917: 26. As a synonym of Balta in Beccaloni 2014.
Allactina Hebard, 1929: 18 (nec Allactina Curran, 1924). Type species: Allactina jacobsoni Hebard, 1929. Synonymized with Balta by Princis 1969: 968.
Graptoblatta Hebard, 1929: 23. Type species: Blatta notulata Stål, 1860. Princis 1969: 957 (as a synonym of Lupparia). Synonymized with Balta by Princis 1969: 968.

#### Diagnosis.

See Che et al. (2010).

#### Discussion.

The characters of *L. nodigera* Bey-Bienko and *L. valida* Bey-Bienko were compared with the generic diagnosis of *Lupparia*: the hind wing of the two species with the apical triangle small or not distinct differs from the main generic characters of *Lupparia*, and are consistent with *Balta*. The two species also share the following characters: 1) abdominal tergites unspecialized, tarsal claws strongly asymmetrical and unspecialized; 2) median phallomere appendage present and with brush-like structure; 3) front femur type B or C, which are also consistent with *Balta*. Therefore the two species are transferred to the genus *Balta*.

#### Checklist of *Balta* species from China

*B. barbellata* Che & Chen, 2010 China (Hainan)

*B. curvirostris* Che & Chen, 2010 China (Hainan)

*B. crena* sp. n. China (Yunnan)

*B. dissecta* Che & Wang, 2010 China (Fujian)

*B. hwangorum* Bey-Bienko, 1958 China (Yunnan)

*B. jinlinorum* Che & Wang, 2010 South China

*B. maculata* sp. n. China (Yunnan)

*B. nodigera* (Bey-Bienko, 1958), comb. n. China (Yunnan)

*B. notulata* Stål, 1860 (Oriental region islands, Indian Ocean islands, Korean Peninsula, Australasian islands)

*B. spinea* Che & Chen, 2010 China (Hainan)

*B. spinescens* Che & Wang, 2010 (Southeast China)

*B. tangi* sp. n. China (Yunnan)

*B. valida* (Bey-Bienko, 1958), comb. n. China (Yunnan)

*B. vilis* Brunner von Wattenwyl, 1865 (Southeast Asia, East Asia)

*B. yaoi* sp. n. China (Yunnan)

#### Key to species of *Balta* from China (males)

**Table:** 

1	Tegmina with round black spots (Figs 3, 7)	**2**
–	Tegmina without black spots (Figs 1, 5, 9, 11, 13)	**3**
2	Median phallomere appendage simple, arched, and without brush-like structure (Fig. 62)	***B. yaoi* sp. n.**
‒	Median phallomere appendage with brush-like structure (Fig. 38)	***B. maculata* sp. n.**
3	Vertex with dark stripes or bands	**4**
‒	Vertex unicolored, without stripes (Figs 40, 64, 76, 88)	**5**
4	Styli triangular, median phallomere long, sticklike, with blunt base, and bifurcated near apex, one arched appendage present	***B. spinea***
‒	Styli elliptical, with scattered fine spines, median phallomere long, sticklike and curved with base blunt and apex brush-like, one arched appendage with apex and base brushlike	***B. spinescens***
5	Styli globular	***B. notulata***
‒	Styli finger-like or conical (Figs 23, 48, 72, 84, 96)	**6**
6	Face black (Fig. 10)	***B. hwangorum***
‒	Face brownish yellow (Figs 2, 6, 12, 14), or with brown stripes (Figs 40)	**7**
7	Styli arising on the inner side of lateral lobes of subgenital plate (Fig. 96)	**8**
‒	Styli arising on the apex of lateral lobes of subgenital plate (Figs 24, 48, 84)	**12**
8	Front femur type B_2_ or B_3_ (Fig. 93)	**9**
‒	Front femur type C_2_ or C_3_ (Figs 45, 81)	**11**
9	Front femur type B_2_	***B. jinlinorum***
‒	Front femur type B_3_ (Fig. 93)	**10**
10	Styli short, the posterior part of subgenital plate strongly protruding in the middle and with trapezoid shape	***B. valida* comb. n.**
‒	Styli short, the posterior part of subgenital plate arced in the middle of emargination but without trapezoid shape	***B. barbellata***
11	Face without stripes (Fig. 76), front femur type C2 (Fig. 81)	***B. nodigera* comb. n.**
‒	Face with stripes (Fig. 40), front femur type C3 (Fig. 43)	***B. tangi* sp. n.**
12	Subgenital plate with spines (Figs 23, 24)	**13**
‒	Subgenital plate without spines	**14**
13	Hind margin of supra-anal plate with U-shape concavity medially (Fig. 22), the apex of median phallomere with some long setae (Fig. 26)	***B. crena* sp. n.**
‒	Hind margin of supra-anal plate broadly rounded, the apex of median phallomere with some long setae	***B. dissecta***
14	Pronotum length 3.0–3.8mm	***B. curvirostris***
‒	Pronotum length 1.9–2.0mm	***B. vilis***

### Balta crena sp. n.

Taxon classificationAnimaliaBlattodeaEctobiidae

http://zoobank.org/8E6159A0-9A4C-4CF7-BBF3-D3243E37B559

Figs 1 , 2 , 15–27

#### Type material.

**Holotype: China**, Yunnan: male (IESWU), Xishuangbanna, Menglun Botanical Garden, Lvshilin, 640 m, 21°54.600′N, 101°17.084′E, 17 November 2009, coll. Guo Tang and Zhiyuan Yao. **Paratypes**: 1 male, same collection event as holotype; 10 males and 14 females, Xishuangbanna, Menglun, G213 (National road) secondary forest, 644 m, 21°54.439′N, 101°17.755′E, 20 November 2009, coll. Guo Tang and Zhiyuan Yao; 4 males and 1 female, Mengla County, Bubeng monsoon forest, 690 m, 21.61379°N, 101.58079°E, 10 August 2012, coll. Guo Zheng, Xue Li and Wenyue Zhu.

#### Differential diagnosis.

This species resembles *Balta notulata* (Stål, 1860) in appearance, but can be distinguished from the latter by the following characters: 1) hind margin of subgenital plate concave and without produced medial lobe (Fig. 24) while that in *B. notulata* with produced medial lobes; 2) some fine spines present on either apex of lateral lobes of subgenital plate (Fig. 24), which are lacking in *B. notulata*.

#### Description.

**Figures 1–14.**
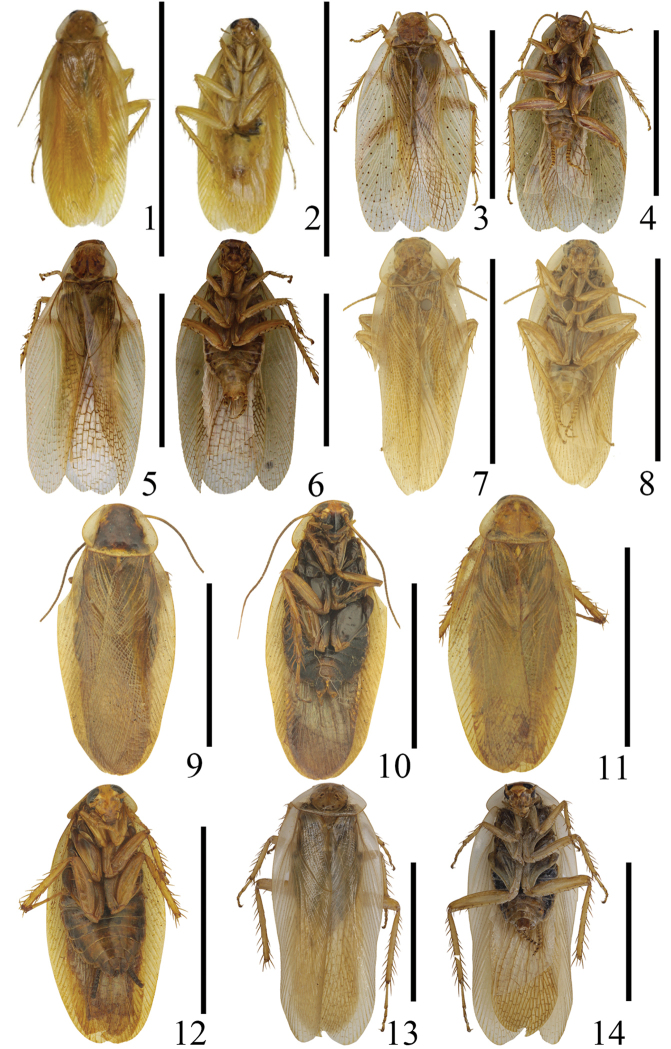
**1–2** *Balta crena* sp. n., male: holotype **1** dorsal view **2** ventral view **3–4** *Balta maculata* sp. n., male: holotype **3** dorsal view **4** ventral view **5–6** *Balta tangi* sp. n., male: holotype **5** dorsal view **6** ventral view **7–8** *Balta yaoi* sp. n., male: holotype **7** dorsal view **8** ventral view **9–10** *Balta hwangorum* Bey-Bienko, 1958, male **9** dorsal view **10** ventral view **11–12** *Balta nodigera* (Bey-Bienko, 1958) comb. n., male **11** dorsal view **12** ventral view **13–14** *Balta valida* (Bey-Bienko, 1958), comb. n., male **13** dorsal view **14** ventral view.

***Male*.** Body brownish yellow (Figs 1, 2). Third and fourth maxillary palpomeres almost the same length, both distinctly longer than the fifth (Fig. 16). Tegmen with M and CuA oblique (Fig. 18); hind wing with M simple; CuA with three or four complete branches and without incomplete ones (Fig. 19). Front femur type C_2_ (Fig. 20), tarsal claws strongly asymmetrical and unspecialized (Fig. 21). Abdominal tergites unspecialized.

**Figures 15–27.**
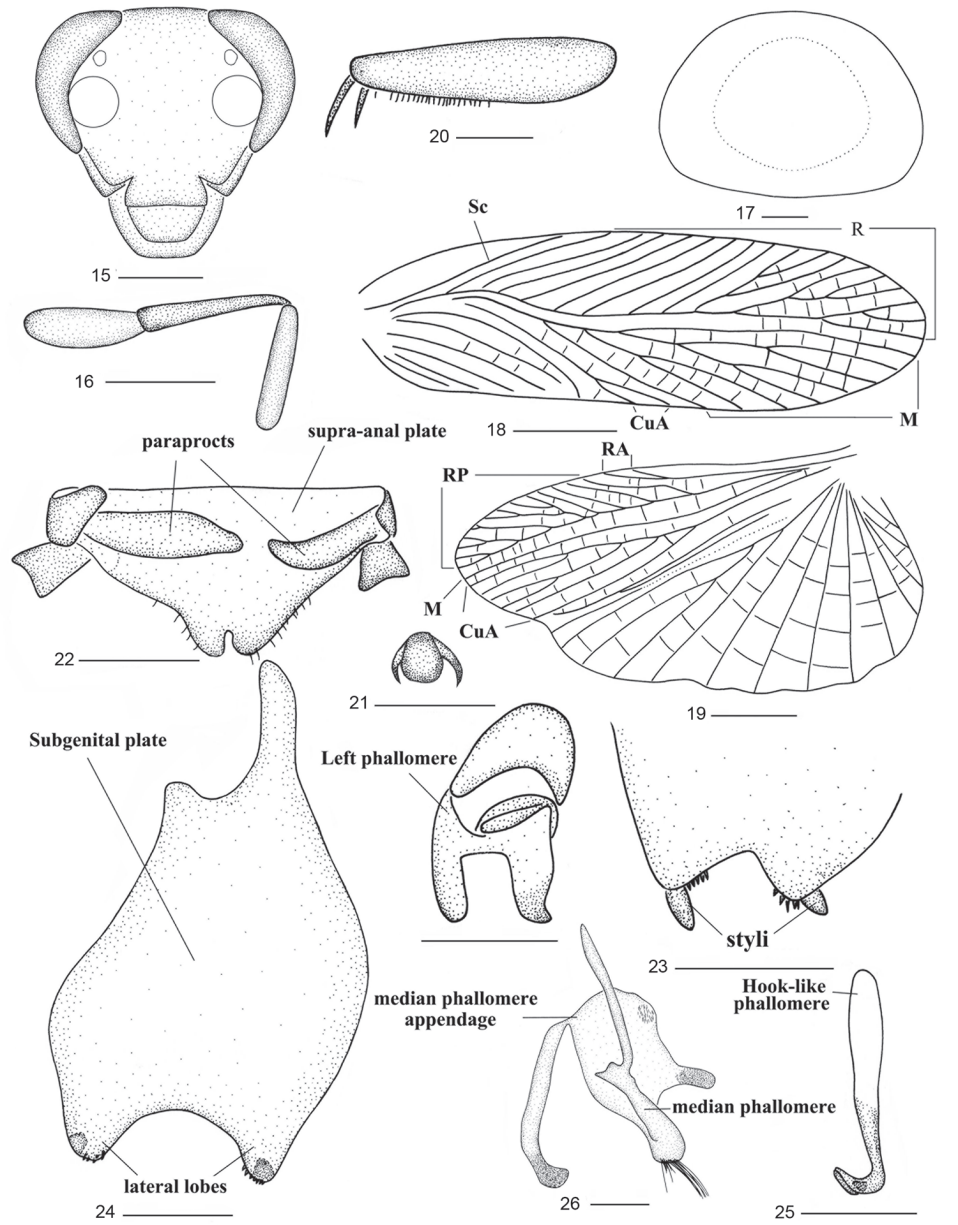
*Balta crena* sp. n. **15** head **16** maxillary palps **17** pronotum **18** tegmen **19** hind wing **20** front femur **21** tarsal claws and arolium **22** supra-anal plate and paraprocts, ventral view **23** subgenital plate, ventral view **24** subgenital plate, dorsal view **25** right phallomere **26** median phallomere **27** left phallomere. Scale bars: 0.5 mm (**15–17, 20–27**), 2.0 mm (**18, 19**).

***Male genitalia*.** Supra-anal plate with posterior margin distinctly produced and concave in U-shape in the middle, right and left paraprocts simple (Fig. 22). Subgenital plate with hind margin strongly concave medially; two lateral lobes with styli on either apex direct dorsally and with fine spines (Figs 23, 24). Hook-like phallomere on right side and with preapical concavity (Fig. 27). Median phallomere with base acuminate, apex blunt with some long setae, median phallomere appendage with some fine spines (Fig. 26). Left phallomere complex, consisted of several irregular sclerites (Fig. 27).

***Female*.** Both tegmina and hind wings slightly beyond the end of abdomen. Subgenital plate with hind margin rounded.

#### Measurements (mm).

Overall length: male 13.5–14.0, female 9.5–10.0; tegmen length male 11.0–12.0, female 7.9–8.2; pronotum length × width male 2.5–2.7 × 3.5–3.8, female 2.5–2.7 × 3.4–3.7.

#### Etymology.

Latin word *crena* means “nick”, referring to subgenital plate with its hind margin strongly concave medially.

#### Distribution.

China (Yunnan).

### Balta maculata sp. n.

Taxon classificationAnimaliaBlattodeaEctobiidae

http://zoobank.org/AD8E7309-BB79-4AF2-915D-9FECA65EAB6E

Figs 3 , 4 , 28–39

#### Type material.

**Holotype: China**, Yunnan: male (IESWU), Xishuangbanna, Menglun, 21 November 2009, coll. Guo Tang. **Paratypes**: 5 males and 5 females, Xishuangbanna, Menglun, 21 November 2009, coll. Guo Tang; 35 males and 63 females, Xishuangbanna, Menglun, G213 (National road) bamboo forest, 581 m, 21°53.622′N, 101°16.955′E, 26 November 2009, coll. Guo Tang and Zhiyuan Yao; 14 males and 50 females, Xishuangbanna, Menglun, G213 (National road) bamboo forest, 2–26 November 2009, coll. Guo Tang and Zhiyuan Yao; 9 males and 12 females, Xishuangbanna, Menglun Botanical Garden, Lvshilin, 652 m, 21°54.710′N, 101°16.941′E, 16 November 2009, coll. Guo Tang and Zhiyuan Yao.

#### Differential diagnosis.

This species resembles *Balta yaoi* sp. n. in appearance, but can be distinguished from the latter by the following characters: 1) tegmen with more obvious black spots, cells slightly more hyaline and without yellowish brown spots (Fig. 31); while in the latter species, the tegmen has fewer and less obvious black spots, cell with yellowish brown spots; 2) median phallomere complex, the appendage base with brush-like structure (Fig. 38); while the latter with median phallomere simple, the appendage arched and without brush-like structure.

**Figures 28–39.**
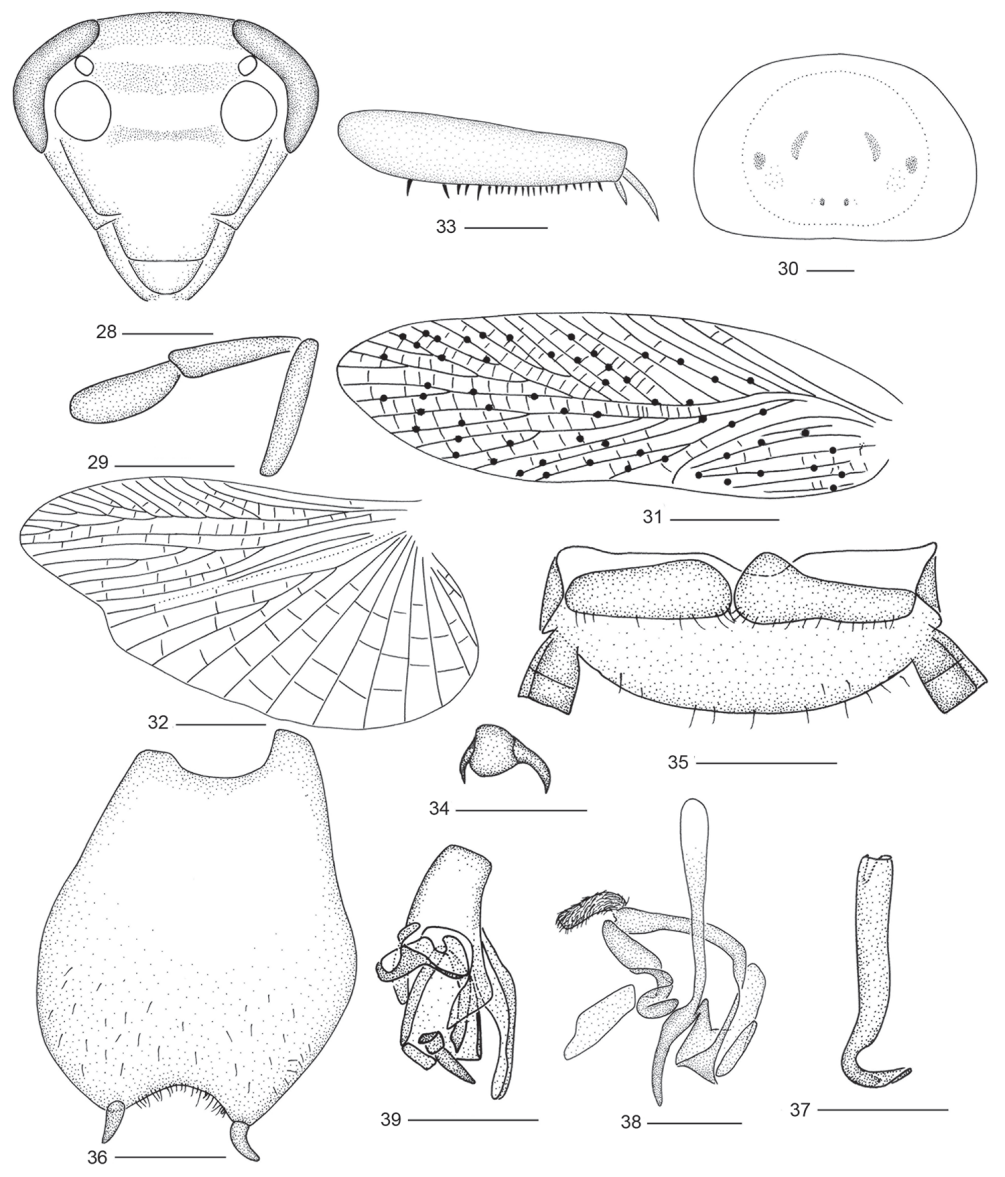
*Balta maculata* sp. n. **28** head **29** maxillary palps **30** pronotum **31** tegmen **32** hind wing **33** front femur **34** tarsal claws and arolium **35** supra-anal plate and paraprocts, ventral view **36** subgenital plate, dorsal view **37** right phallomere **38** median phallomere **39** left phallomere. Scale bars: 0.5 mm (**28–30, 33–39**), 2.0 mm (**31, 32**).

#### Description.

**Male.** Body tawny. Face between eyes brown, between ocelli and antenna sockets with a light brown stripe, under antennal sockets also with a light brown stripe (Fig. 28). Pronotum with some small scattered and symmetrical spots or inconspicuous stripes, with tawny disk, lateral borders hyaline (Fig. 30). Tegmen and hind wing tawny, tegmen with black spots (most distributed on veins) (Fig. 31). The middle of anterior margin of abdominal tergites blackish brown. Third and fourth maxillary palpomeres approximately same length, both distinctly longer than the fifth (Fig. 29). Tegmen with M and CuA oblique (Fig. 31). CuA of hind wing with three complete branches and without incomplete ones (Fig. 32). Front femur of type C_2_ (Fig. 33), tarsal claws strongly asymmetrical and unspecialized (Fig. 34). Abdominal tergites unspecialized.

**Male genitalia.** Supra-anal plate short, hind margin rounded; right and left paraprocts similar (Fig. 35). Subgenital plate with hind margin strongly concaved; styli conical, arising on apexes of lateral lobes and slightly curved laterally (Fig. 36). Hook-like phallomere on the right side and with preapical concavity (Fig. 37). Median phallomere stick-like, curved near acuminated apex, median phallomere appendage present and with brush-like structure (Fig. 38). Left phallomere complex (Fig. 39).

**Female.** Supra-anal plate longer than subgenital plate, hind margin of the former with triangular process in the middle. Subgenital plate simple with hind margin rounded.

#### Measurements (mm).

Overall length male 14.0–15.0, female 9.8–10.2; tegmen length male 12.1–12.5, female 8.0–8.5; pronotum length × width male 2.4–2.9 × 3.4–3.8, female 2.5–3.0 × 3.6–4.0.

#### Etymology.

Latin word *maculata* meaning “with dots”, referring to the tegmina with dots.

#### Distribution.

China (Yunnan).

### Balta tangi sp. n.

Taxon classificationAnimaliaBlattodeaEctobiidae

http://zoobank.org/511EDAE2-8FC8-48CC-8A94-FAE64D43E411

Figs 5 , 6 , 40–51

#### Type material.

**Holotype: China**, Yunnan: male (IESWU), Xishuangbanna, Menglun Botanical Garden, Lvshilin, 652 m, 21°54.710′N, 101°16.941′E, 16 November 2009, coll. Guo Tang and Zhiyuan Yao. **Paratypes**: 25 males and 16 females, same collection event as holotype; 8 males and 8 females, Xishuangbanna, Menglun, G213 (National Road) bamboo forest, 21–26 November 2009, coll. Guo Tang and Zhiyuan Yao; 11 males and 21 females, Xishuangbanna, Menglun Botanical Garden, Lvshilin, 656 m, 21°54.705′N, 101°16.898′E, 13 November 2009, coll. Guo Tang and Zhiyuan Yao.

#### Differential diagnosis.

**Figures 40–51.**
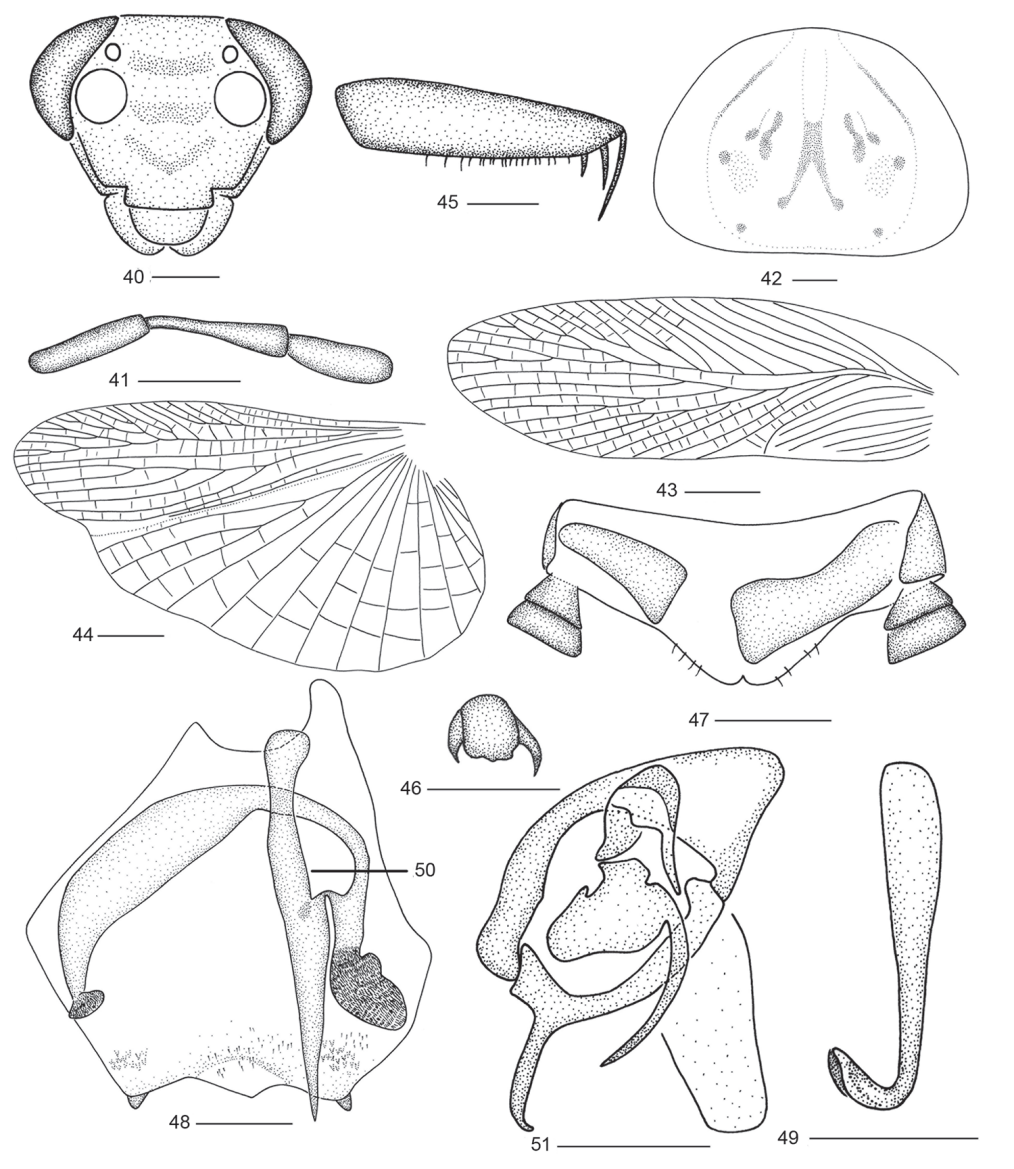
*Balta tangi* sp. n. **40** head **41** maxillary palps **42** pronotum **43** tegmen **44** hind wing **45** front femur; **46** tarsal claws and arolium **47** supra-anal plate and paraprocts, ventral view **48** subgenital plate, dorsal view **49** right phallomere **50** median phallomere **51** left phallomere; Scale bars: 0.5 mm (**40–42, 45–51**), 2.0 mm (**43, 44**).

*Balta tangi* species resembles *B. spinea* in appearance, but can be distinguished from the latter by the following characters: 1) face with transversal stripes between interocular space (Fig. 40) while face of *B. spinea* without transversal stripes but having two longitudinal stripes, each of them with one small rounded white spot; 2) subgenital plate in ventral view between the styli slightly emarginated and with a protrusion in the middle (Fig. 48) but in *B. spinea* without the protrusion.

#### Description.

**Male.** Body yellowish brown. Vertex brownish yellow. Face with three stripes (Fig. 40). Disk of pronotum yellowish brown and with some small scattered and symmetrical blackish brown spots, lateral borders of pronotum hyaline (Fig. 42). Lateral border of abdomen sterna with round blackish brown spots (Fig. 6). Third and fourth maxillary palpomeres nearly the same length, distinctly longer than the fifth (Fig. 41). Hind wing with triangle apical, near apex of R with posterior branches, CuA with three complete branches (Fig. 44). Front femur type C_3_ (Fig. 45), tarsal claws strongly asymmetrical and unspecialized (Fig. 46). Abdominal tergites unspecialized.

**Male genitalia.** Supra-anal plate symmetrical with hind margin slightly concave in the middle, paraprocts simple (Fig. 47). Subgenital plate with hind margin slightly emarginated and with process in the middle, conical styli located on the apex of either lateral lobes respectively, ventral side of subgenital plate with some fine spines near the stylus (Fig. 48). Hook-like phallomere on right side and with preapical concavity (Fig. 49). Median phallomere sticklike, base thick and strong, apex gradually becomes sharp-pointed, near the middle part of median phallomere with an arc-shaped appendage, base, and apex with short setae (Fig. 50). Left phallomere complex, apex with spinous structure (Fig. 51).

**Female.** Sexual dimorphism, female body thicker and stronger than male, both tegmina and hind wings degraded, just extending to supra-anal plate, RA with one or two branches.

#### Measurements (mm).

Overall length male 14.5–16.0, female 12.0–13.5; tegmen length male 13.5–14.0, female 9.4–9.6; pronotum length × width male 2.9–3.0 × 3.9–4.1, female 2.8–3.0 × 3.9–4.2.

#### Etymology.

This species is named in honor of Mr. Guo Tang, who collected the holotype of the species.

#### Distribution.

China (Yunnan).

### Balta yaoi sp. n.

Taxon classificationAnimaliaBlattodeaEctobiidae

http://zoobank.org/AA25257D-4E10-4C22-99EA-4808E7FF794A

Figs 7–8 , 52–63

#### Type material.

**Holotype: China**, Yunnan: male (IESWU), Xishuangbanna, Menglun, 2010, coll. Zhiyuan Yao. **Paratypes**: 4 males, same collection event as holotype; 14 males and 22 females, Xishuangbanna, Menglun, garbage dump, 627 m, 21°54.380′N, 101°16.815′E, 23 November 2009, coll. Guo Tang and Zhiyuan Yao; 3 males, Xishuangbanna, Menglun, 2010, coll. unknown;

#### Differential diagnosis.

This species is similar to *B. valida* comb. n. in appearance, but can be distinguished in the following characteristics: 1) For the former, median phallomere appendage without brush-like structure (Fig. 62), while in the latter with brush-like structure; 2) subgenital plate with hind margin curved concave (Fig. 60), while in the latter not concaved medially.

**Figures 52–63.**
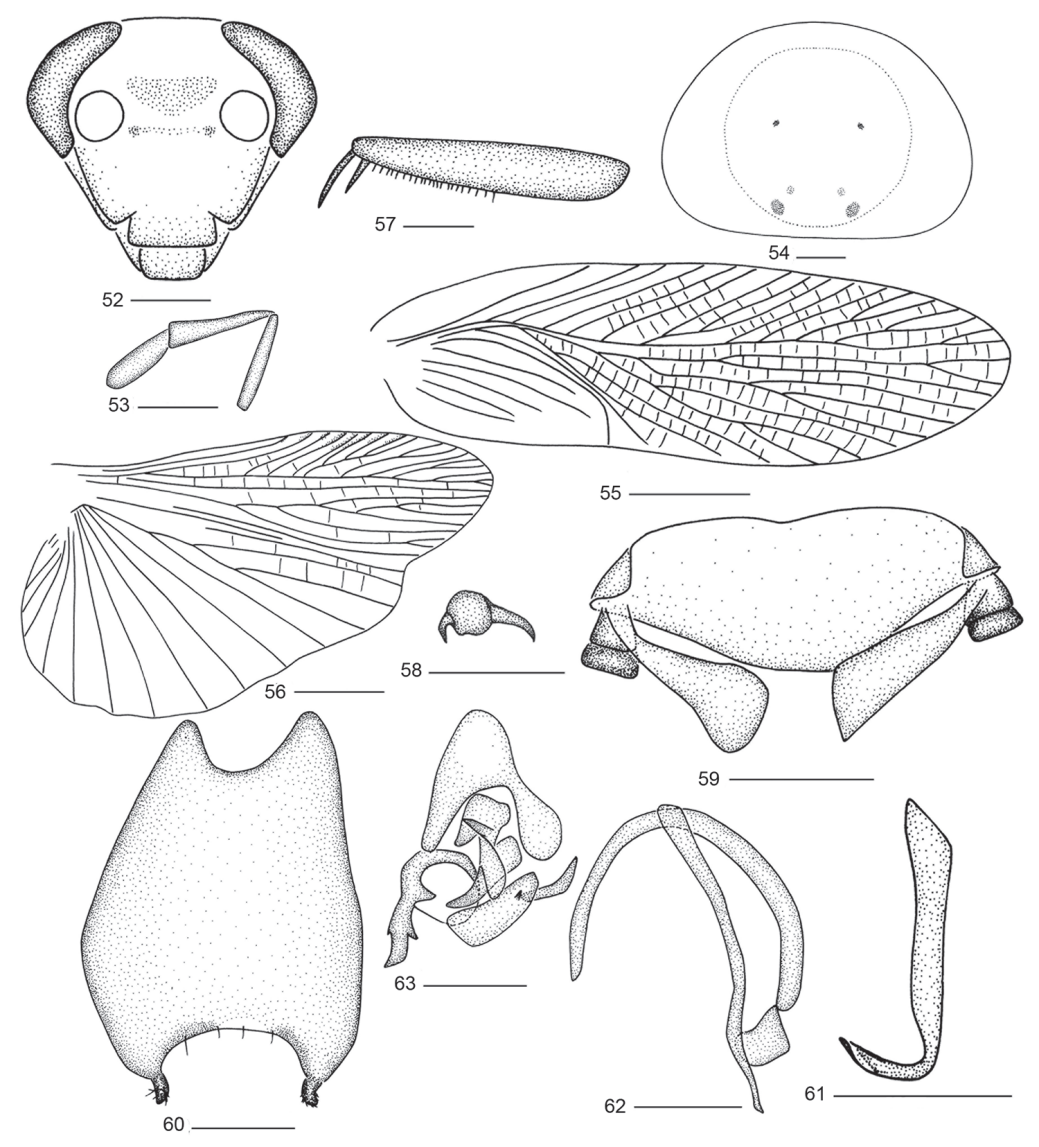
*Balta yaoi* sp. n. **52** head **53** maxillary palps **54** pronotum **55** tegmen **56** hind wing **57** front femur **58** tarsal claws and arolium **59** supra-anal plate and paraprocts, ventral view **60** subgenital plate, dorsal view **61** right phallomere **62** median phallomere **63** left phallomere. Scale bars: 0.5 mm (**52–54, 57–63**), 2.0 mm (**55, 56**).

**Description male.** Body yellowish brown (Figs 7, 8). Vertex slightly yellow, between eyes with a slightly brown stripe, under the stripe with a slightly brown triangular spot, under antennal sockets with or without a stripe (Fig. 52). Disk of pronotum yellowish brown, with some small scattered and symmetrical blackish brown spots, two lateral border hyaline (Fig. 54). Tegmen yellowish brown, with a few small round black spots, cell with yellowish brown spots. The lateral border of abdomen with round blackish brown spots. Third and fourth maxillary palpomeres nearly same length, longer than the fifth (Fig. 53). Tegmen with M and CuA oblique (Fig. 55). Hind wing with M simple, without branches, CuA with three complete branches and without incomplete ones, hind wing with small apical triangle (Fig. 56). Front femur Type C_2_ (Fig. 57), tarsal claws strongly asymmetrical and unspecialized (Fig. 58). Abdominal tergites unspecialized.

**Male genitalia.** Supra-anal plate short, hind margin finely rounded, right and left paraprocts slightly unsymmetrical (Fig. 59). Subgenital plate with hind margin strongly emarginated, styli located on the apex of each lateral lobe respectively and slightly curved laterally (Fig. 60). Hook-like phallomere on the right side and with preapical concavity (Fig. 61). Median phallomere sticklike, slightly curved, apex acuminate, median phallomere appendage arched (Fig. 62). Left phallomere complex, without brush-like structure (Fig. 63).

**Female.** Both tegmina and hind wings slightly beyond the end of abdomen. The end of abdomen rounded.

#### Measurements (mm).

Overall length of male 12.0–13.0, female 10.0–10.3; tegmen length male 10.0–11.0, female 7.9–8.0; pronotum length × width male 2.3–2.6 × 3.4–3.6, female 2.2–2.5 × 2.8–3.0.

#### Etymology.

This species is named in honor of Mr. Zhiyuan Yao, who collected the holotype of the species.

#### Distribution.

China (Yunnan).

### Balta hwangorum

Taxon classificationAnimaliaBlattodeaEctobiidae

Bey-Bienko, 1958

Figs 9 , 10 , 64–75

Balta hwangorum Bey-Bienko, 1958: 676, 688 (Type locality: Yunnan, China); Princis 1969: 978.
Balta picea Bey-Bienko, 1958: 677 (Holotype, female) (Type locality: Yunnan, China). Syn. n.

#### Material examined.

Deposited in IESWU. **China**, Yunnan Prov.: 3 males, Xishuangbanna, Damenglong, 650 m, 13 April 1958, coll. Zhizi Chen; 5 males, Xishuangbanna, Menghun, 650–750 m, 9 June 1958, coll. Xuwu Meng; 1 male and 1 female, Xishuangbanna, Jinghong, 650 m, 6–24 July 1958, coll. Junhua He; 1 male, Xishuangbanna, Menghun, 650–750m, 1 June 1958, coll. Leyi Zheng; 1 male, Xishuangbanna, Menghun, 650–1080 m, 7 June 1958, coll. Chunpei Hong and Shuyong Wang; 1 male, Xishuangbanna, Damenglong, 650 m, 14 April 1958, coll. Chunpei Hong; 1 male, Xishuangbanna, Menghun, 650–750 m, 3 February 1958, coll. Shuyong Wang; 2 males, Xishuangbanna, Menga, 1050–1080 m, 20 May 1958, coll. Fuji Pu; 1 male, Xishuangbanna, Menghun, 650–750 m, 13 June 1958, coll. Yiran Zhang; 1 male, Xishuangbanna, Menghun, 650–750 m, 18 April 1958, coll. Leyi Zheng; 1 male, Xishuangbanna, Xiaomengyang, 850 m, 13 June 1958, coll. Lingchao Zang; 2 males and 1 female, Xishuangbanna, Menglun, G213 (National road) bamboo forest, 627 m, 21°54.380′N, 101°16.815′E, 21–26 November 2009, coll. Guo Tang and Zhiyuan Yao; 2 males and 2 females, Xishuangbanna, Menglun, G213 (National road) bamboo forest, 627 m, 21°54.380′N, 101°16.815′E, 22 November 2009, coll. Guo Tang and Zhiyuan Yao; 2 males and 2 females, Xishuangbanna, Menglun, G213 (National road) secondary forest, 644 m, 21°54.439′N, 101°16.755′E, 20 November 2009, coll. Guo Tang and Zhiyuan Yao; 1 male, Mojiang, 1 May 2013, coll. Zongqing Wang. China: Guangxi: 1 female, Pingxiang, 850 m, 11 May 1963, coll. Sikong Liu.

#### Redescription.

**Male.** Body yellowish brown (Figs 9, 10). Vertex to anterior margin of antennal sockets reddish brown, face yellowish brown to blackish brown (Figs 9, 10). Pronotum with inconspicuous black brindle or two oblique and symmetrical stripes, with disk reddish brown to black brown, two lateral borders, anterior and posterior margin yellowish brown and hyaline (Fig. 66). Legs yellowish brown to blackish brown. Abdomen blackish brown. Apex of subgenital plate with reddish brown spots or without. Fourth and fifth maxillary palpomeres approximately same length, both distinctly shorter than the third (Fig. 65). Tegmen with M and CuA slightly oblique (Fig. 67). Hind wing with M without branches, CuA with five complete branches and without incomplete ones (Fig. 68). Front femur type C_2_ (Fig. 69), tarsal claws strongly asymmetrical and unspecialized (Fig. 70). Abdominal tergites unspecialized.

**Figures 64–75.**
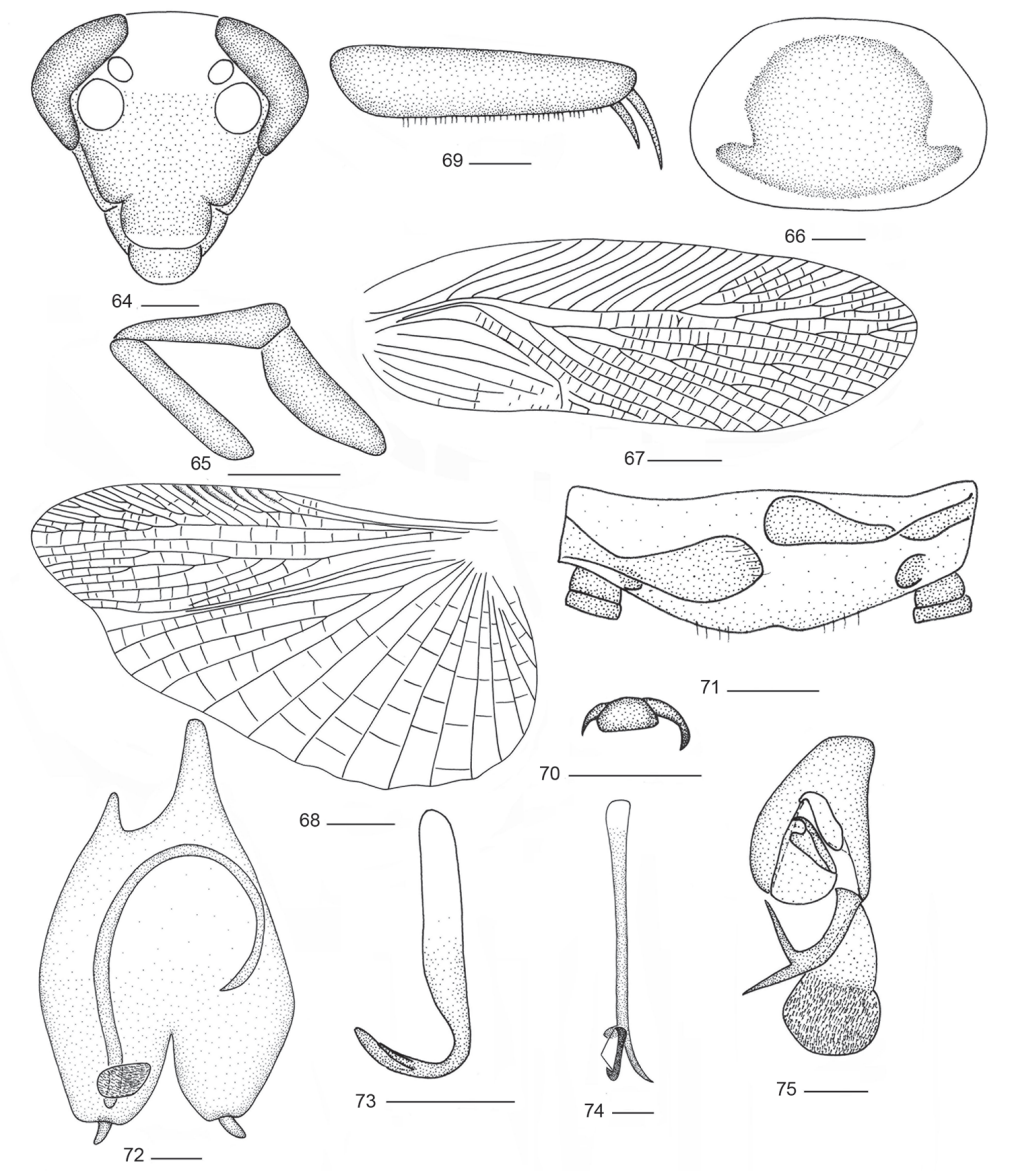
*Balta hwangorum* Bey-Bienko, 1958. **64** head **65** maxillary palps **66** pronotum; **67** tegmen **68** hind wing **69** front femur **70** tarsal claws and arolium **71** supra-anal plate and paraprocts, ventral view **72** subgenital plate, dorsal view **73** right phallomere **74** median phallomere **75** left phallomere. Scale bars: 0.5 mm (**64–66, 69–75**), 2.0 mm (**67, 68**).

#### Male genitalia.

Supra-anal plate in ventral view short, hind margin arched, slightly emarginated in the middle; right and left paraprocts similar (Fig. 71). Subgenital plate symmetrical throughout except at the base, hind margin with deep V-shape emargination, styli similar, conical, arising on lateral lobes (Fig. 72). Hook-like phallomere on right side, hook slender (Fig. 73). Median phallomere sticklike, slightly curved, apex acuminate, near the apex part with a sclerite, base of arc appendage with brush-like structure (Fig. 74). Left phallomere complex, apex with a bifurcate spinous structure and a slender hairs structure (Fig. 75).

#### Female.

Slight sexual dimorphism in that the female body is smaller than the male. Supra-anal plate symmetrical, rounded, hind margin divided in the middle. Subgenital plate broad and rounded.

#### Measurement (mm).

Overall length male 15.0–18.5, female 14.0–17.1; tegmen length male 13.5–16.0, female 11.2–14.0; pronotum length × width male 2.9–3.5 × 4.1–4.9, female 3.0–3.1 × 5.0–5.1.

#### Remarks.

Several specimens were separated as two species by Bey-Bienko, mainly based on the body color (1958). However, after examining a large number of specimens, the results show that body color of *B. hwangorum* varies. Moreover, in Bey-Bienko’s records, in *B. hwangorum*, the length of lobes of the subgenital plate is different among the species, but in fact we find no difference.

#### Distribution.

China (Yunnan, Guangxi).

### Balta nodigera

Taxon classificationAnimaliaBlattodeaEctobiidae

(Bey-Bienko, 1958) comb. n.

Figs 11 , 12 , 76–87

Onychostylus nodiger Bey-Bienko, 1958: 679 (Type locality: Yunnan, China).
Lupparia nodigera : Princis 1969: 960.

#### Material examined.

Deposited in IESWU. **China**, Fujian Prov.: 1 male, Putian, 7 June 1979, coll. Jingying Liu; 1 male, Putian, 5 July 1978, coll. Bangkan Huang.

#### Redescription.

**Male.** Body medium-size, yellowish brown (Figs 11, 12). Third and fourth maxillary palpomeres yellowish brown, the fifth light brown. Pronotum yellowish brown and disk with two unobvious black spots, lateral borders hyaline. Tegmina and hind wings light brown. Abdomen brown. Third and fifth maxillary palpomeres almost same length, distinctly longer (Fig. 77). Tegmen with M and CuA oblique (Fig. 79). Hind wing with small triangle apical, CuA with four complete branches and without incomplete ones (Fig. 80). Front femur type C_2_ (Fig. 81), tarsal claws strongly asymmetrical and unspecialized (Fig. 82). Abdominal tergites unspecialized.

**Figures 76–87.**
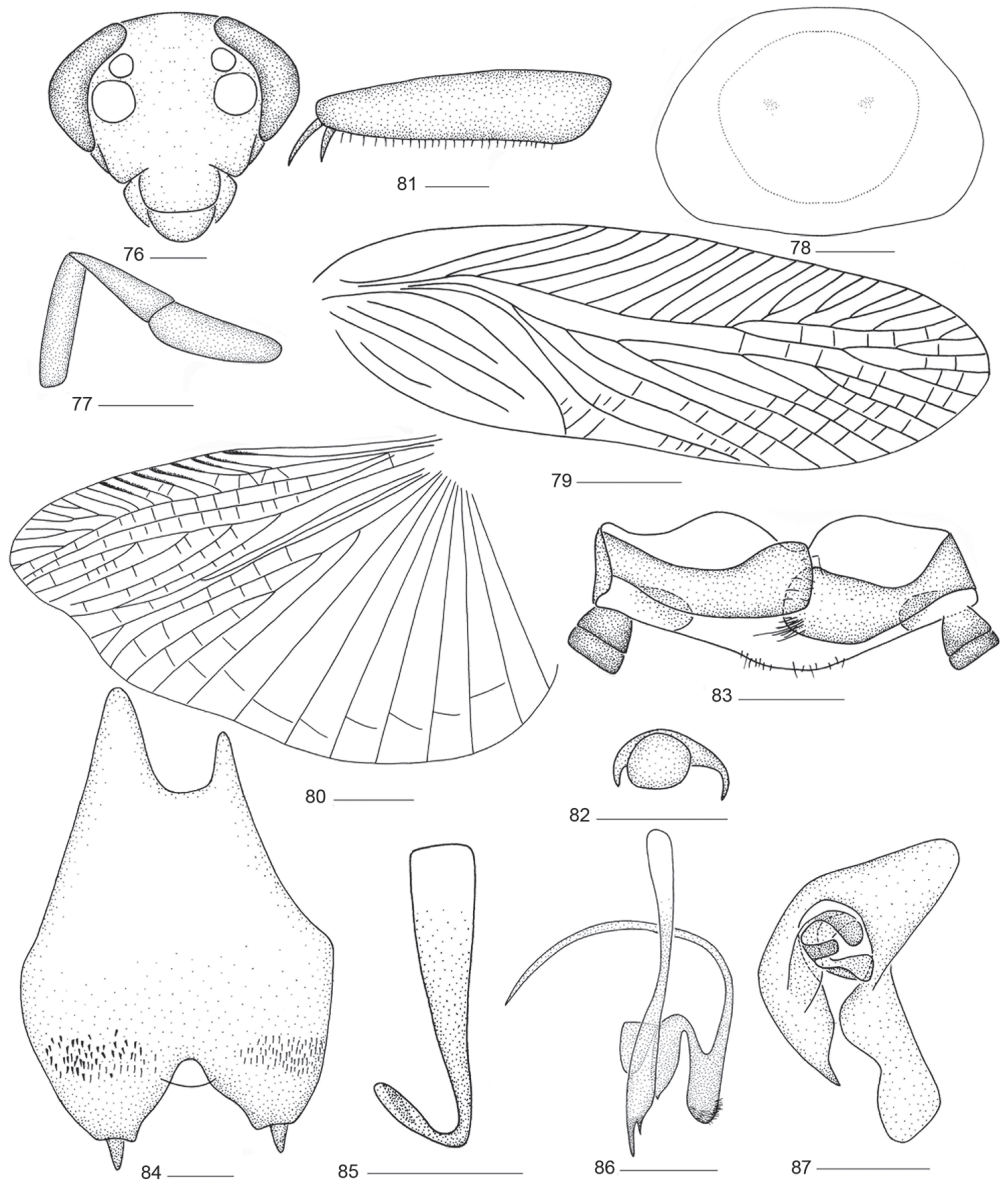
*Balta nodigera* (Bey-Bienko, 1958) comb. n. **76** head **77** maxillary palps **78** pronotum **79** tegmen **80** hind wing **81** front femur **82** tarsal claws and arolium **83** supra-anal plate and paraprocts, ventral view **84** subgenital plate, dorsal view **85** right phallomere **86** median phallomere **87** left phallomere. Scale bars: 0.5 mm (**76–78, 81–87**), 2.0 mm (Figs **79, 80**).

#### Male genitalia.

Supra-anal plate posterior margin slightly convex in the middle, paraprocts simple (Fig. 83). Subgenital plate in ventral view with hind margins strongly emarginated in the middle, conical styli located on the distal of lateral lobes, ventral side of subgenital plate with some fine spines (Fig. 84). Hook-like phallomere on right side, the hook structure short and thick (Fig. 85). Median phallomere sticklike, thick and strong at base, apex with two different size spines; one associated median phallomere appendage stronger and with setae at apex, (Fig. 86). Left phallomere with left side apex spine-like (Fig. 87).

#### Measurements (mm).

Overall length male 14.5; tegmen length male 12.0; pronotum length × width male 3.0 × 4.0.

#### Distribution.

China (Fujian, Yunnan).

### Balta valida

Taxon classificationAnimaliaBlattodeaEctobiidae

(Bey-Bienko, 1958) comb. n.

Figs 13 , 14 , 88–99

Onychostylus validus Bey-Bienko, 1958: 589 (Type locality: Yunnan, China).
Lupparia valida : Princis 1969: 957.

#### Material examined.

Deposited in IESWU. **China**, Yunnan Prov.: 19 males, Xishuangbanna, Menghun, 650 m, 4–9 April 1958, coll. Yiran Zhang and Xuwu Meng; 1 male and 1 female, Xishuangbanna, Dadugang, 22°22.190′N, 100°56.977′E, 29 May 2014, coll. Xinran Li and Hongguang Liu; 5 males and 5 females, Puer, Meizihu park, 20 May 2016, coll. Zhiwei Qiu and Lu Qiu; 2 males and 1 female, Xishuangbanna, Wangtianshu, 22 May 2016 coll. Zhiwei Qiu and Lu Qiu. China, Hainan Prov.: 1 male, Wuzhi Mountain, 18°54.290′N, 109°41.081’E, 795 m, 18 May 2014, coll. Shunhua Gui and Xinran Li. 1 male, Limu Mountain, 16 April 2016, coll. Jianyue Qiu.

#### Redescription.

**Male.** Body yellowish brown (Figs 13, 14). Vertex between eyes brick-red, between two ocelli yellow, remainder of face yellowish brown and without stripes (Figs 13, 14). Maxillary palpomeres yellowish brown with either base blackish brown. Base of antenna yellowish brown and the rest blackish brown to black. Pronotum yellowish brown and disc with some black spots, lateral borders hyaline. Legs yellowish brown. Base and two borders of abdominal sternites black (Fig. 14). Third and fourth maxillary palpomeres approximately same length, both distinctly longer than the fifth (Fig. 89). Tegmen with M degeneration and CuA with more branches (Fig. 91). Hind wing with M without branches, near apex of R with short branches, CuA with four to six complete branches and two or three branches of them bifurcated near apex (Fig. 92). Front femur type B_3_ (Fig. 93), tarsal claws strongly asymmetrical and unspecialized (Fig. 94). Abdominal tergites unspecialized.

**Figures 88–99.**
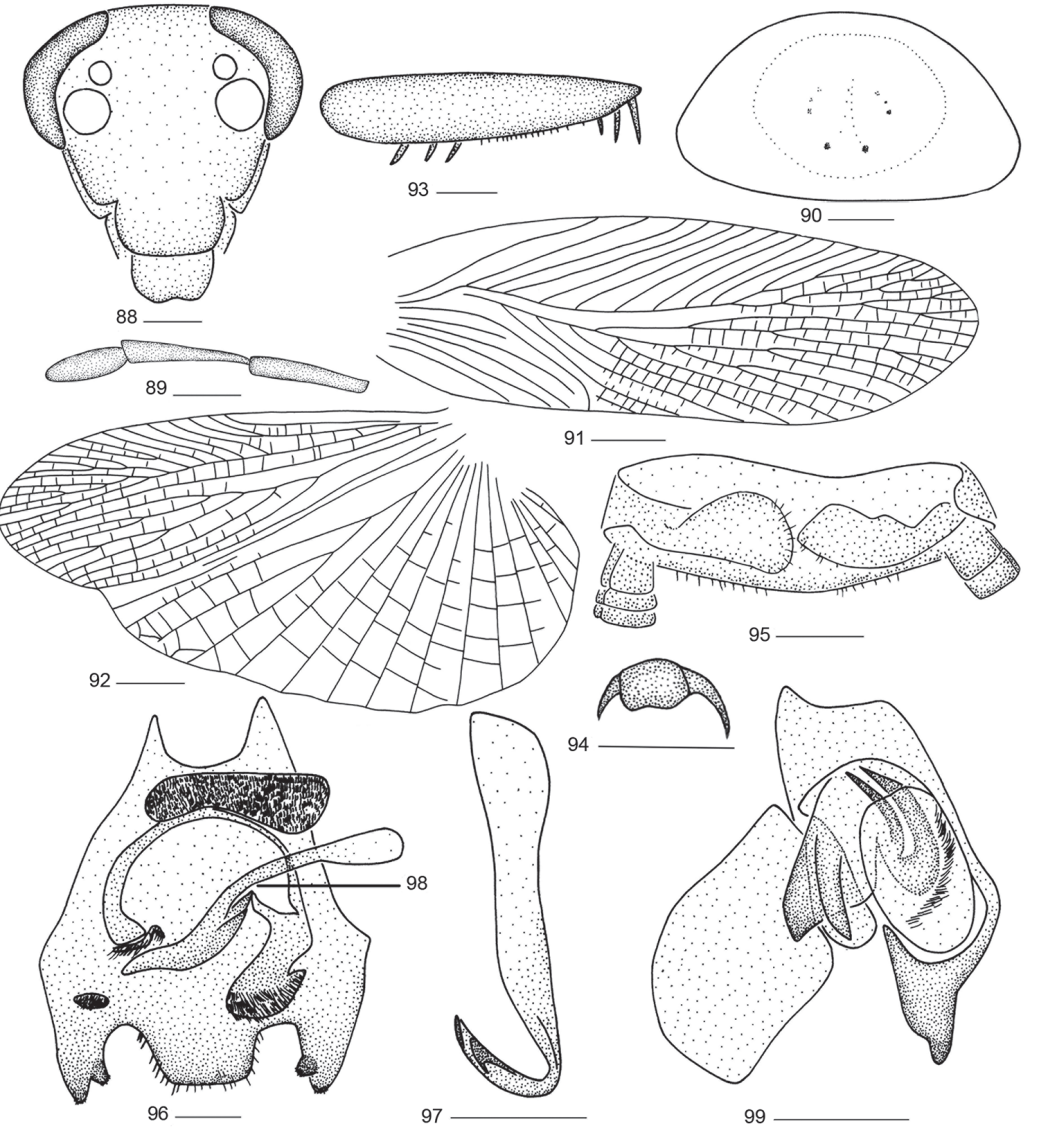
*Balta valida* (Bey-Bienko, 1958) comb. n. **88** head **89** maxillary palps **90** pronotum **91** tegmen **92** hind wing **93** front femur; **94** tarsal claws and arolium; **95** supra-anal plate and paraprocts, ventral view **96** subgenital plate, dorsal view **97** right phallomere **98** median phallomere **99** left phallomere. Scale bars: 0.5 mm (**88–90, 93–99**), 2.0 mm (**91, 92**).

**Male genitalia.** Supra-anal plate in ventral view short, hind margin finely rounded, right and left paraprocts unsymmetrical (Fig. 95). The hind margin of subgenital plate concave near lateral sides and with densely setae; styli conical, with densely scattered short setae, located on lateral lobes (Fig. 96). Hook-like phallomere on right side and with preapical concavity (Fig. 97). Median phallomere thick and strong and near apex with a brush-like structure; base of appendage also with long strip brush-like structure (Fig. 98). Left phallomere complex, with brush-like structure (Fig. 99).

#### Measurements (mm).

Overall length male 16.3–20.5, female 14.5–17.0; tegmen length male 14.2–17.0, female 12.0–14.1; pronotum length × width male 2.3–4.0 × 3.3–5.3, female 3–4.3 × 3.3–5.1.

#### Distribution.

China (Hainan, Yunnan).

## Supplementary Material

XML Treatment for Balta

XML Treatment for Balta crena

XML Treatment for Balta maculata

XML Treatment for Balta tangi

XML Treatment for Balta yaoi

XML Treatment for Balta hwangorum

XML Treatment for Balta nodigera

XML Treatment for Balta valida
